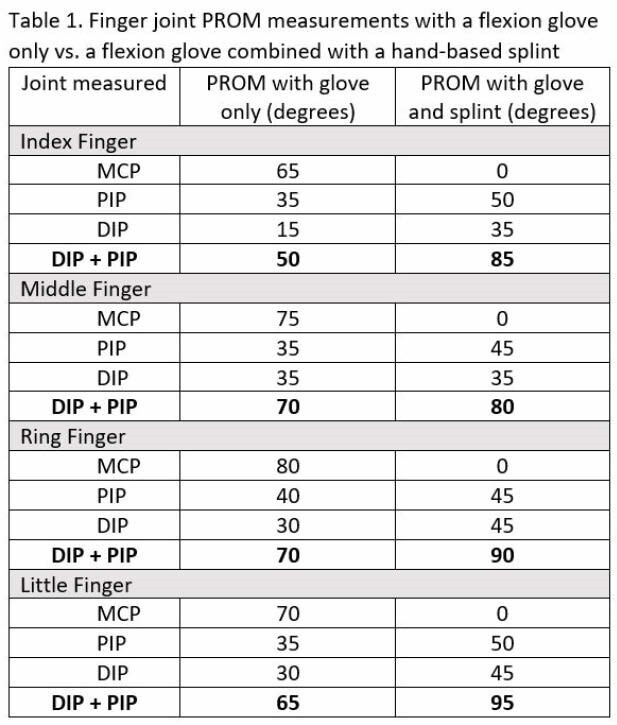# 123 Flexion Glove Modification to Isolate Proximal and Distal Interphalangeal Joint Range of Motion

**DOI:** 10.1093/jbcr/irad045.096

**Published:** 2023-05-15

**Authors:** Joshua Rodriguez, Scott Vocke, Gregory Andre, Brooke Dean

**Affiliations:** Johns Hopkins Bayview Medical Center, Timonium, Maryland; Johns Hopkins Bayview Medical Center, Baltimore, Maryland; Johns Hopkins Bayview Medical Center, Lutherville Timonium, Maryland; Johns Hopkins Bayview Medical Center, Baltimore, Maryland; Johns Hopkins Bayview Medical Center, Timonium, Maryland; Johns Hopkins Bayview Medical Center, Baltimore, Maryland; Johns Hopkins Bayview Medical Center, Lutherville Timonium, Maryland; Johns Hopkins Bayview Medical Center, Baltimore, Maryland; Johns Hopkins Bayview Medical Center, Timonium, Maryland; Johns Hopkins Bayview Medical Center, Baltimore, Maryland; Johns Hopkins Bayview Medical Center, Lutherville Timonium, Maryland; Johns Hopkins Bayview Medical Center, Baltimore, Maryland; Johns Hopkins Bayview Medical Center, Timonium, Maryland; Johns Hopkins Bayview Medical Center, Baltimore, Maryland; Johns Hopkins Bayview Medical Center, Lutherville Timonium, Maryland; Johns Hopkins Bayview Medical Center, Baltimore, Maryland

## Abstract

**Introduction:**

Flexion gloves are a quick and easy way to provide a dynamic stretch to the digits for patients with limited composite flexion range of motion (ROM) or who are at risk for digit extension burn scar contractures. However, the flexion glove has been observed clinically to bias metacarpal phalangeal (MCP) joint flexion ROM when compared to the proximal and distal phalangeal (PIP and DIP) joints. The purpose of this case study is to demonstrate the use of a custom thermoplastic hand-based splint to block MCP joint motion in order to better isolate PIP and DIP joint ROM for a patient with primary PIP and DIP flexion ROM limitations.

**Methods:**

A custom, thermoplastic, palmer hand-based splint was fabricated with an opening for the thumb and the splint extending to the proximal phalanxes stopping below the PIP joints in order to block MCP joint flexion ROM as seen in Figure 1b. Composite flexion passive ROM (PROM) measurements were taken for all MCP, PIP, and DIP joints of digits 2-4 with only the flexion glove donned and with the hand-based splint applied over the flexion glove as seen in Figure 1a and 1c.

**Results:**

An increase in combined PIP and DIP joint PROM was found for all digits when the hand-based splint was donned over the flexion glove compared to the flexion glove only (see Table 1). These increases in combined PIP and DIP PROM included 35° for the index finger, 10° for the middle finger, 20° for the ring finger and 30° for the small finger, with an overall mean increase of 23.75°

**Conclusions:**

This easy splinting technique combined with a flexion glove was effectively used to bias stretch of the PIP and DIP joints into flexion for a patient with adequate MCP flexion ROM and impaired PIP and DIP flexion ROM caused by burn scar contracture.

**Applicability of Research to Practice:**

Using an off the shelf flexion glove and a basic hand-based splint, therapists can use this technique to easily adjust the angle of pull to maximize the benefit from a flexion glove to address specific joint limitations of the finger caused by burn scar contracture.